# Supplementary material for: Regulatory T Cell as a Biomarker of Treatment-Free Remission in Patients with Chronic Myeloid Leukemia
Source: Cancers (Basel). 2021 Nov 24;13(23):5904. doi: 10.3390/cancers13235904 (PMC8657169; doi:10.3390/cancers13235904)
Supplement: Supplementary file 1 [file cancers-13-05904-s001.zip › cancers-1427954-supplementary.pdf]

Supplementary Files

Regulatory T Cell as a Biomarker of Treatment-Free Remission in Patients with Chronic Myeloid Leukemia

Yuki Fujioka, Daisuke Sugiyama, Itaru Matsumura, Yosuke Minami, Masatomo Miura, Yoshiko Atsuta, Shigeki Ohtake, Hitoshi Kiyoi, Yasushi Miyazaki, Hiroyoshi Nishikawa and Naoto Takahashi

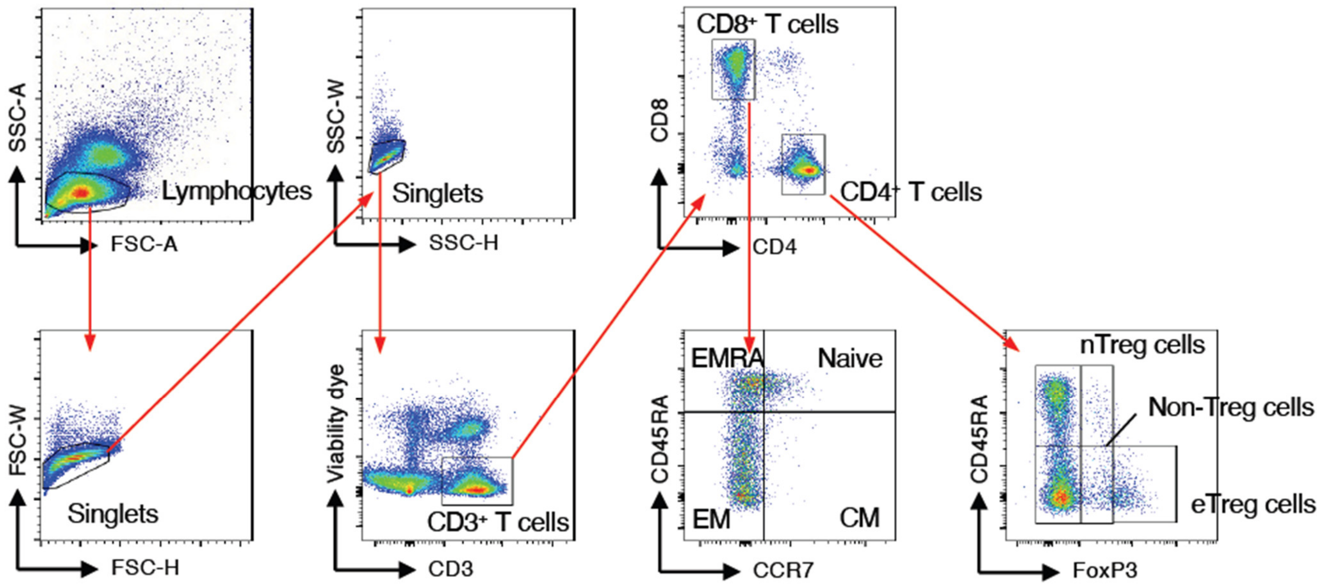

Figure S1. Gating strategy of flow cytometry. Representative plots were shown. CM, central memory. EM, effector memory. EMRA, CD45RA+ EM. eTreg, effector regulatory T.

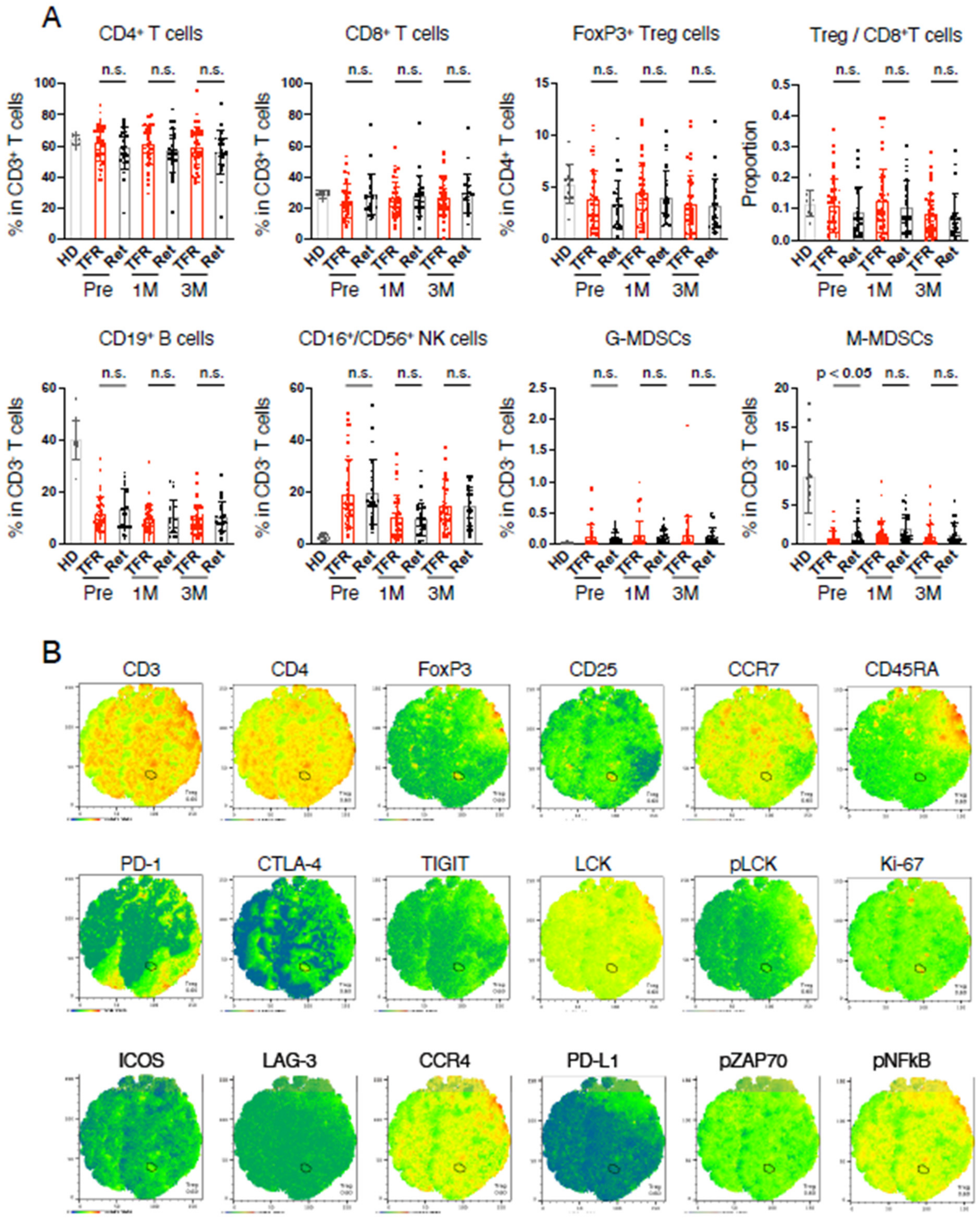

Figure S2. Analysis of PBMCs of chronic myeloid leukemia (CML) patients using mass cytometry. (**A**) Representative *t*-distributed stochastic neighbor embedding (*t*-SNE) plots in lymphocytes (left). Histograms show the expressions of CD16, CD19, CD15, CD3, CD33, CD14, CD11b, CD8, and CD4 among fractions. Sequential *t*-SNE plots of the patient from treatment-free remission (TFR) and retreatment groups before stopping imatinib (referred to as pre-stopping hereafter) and at 1 and 3 months after stopping imatinib administration (Pre, 1M, and 3M, respectively; right). (**B**) Frequencies of each immune cell and regulatory T (Treg) cell/CD8^{+}T ratio in a healthy donor (HD) and in patients with CML from the TFR (red) or retreatment (Ret) (black) groups. (**C**) Representative *t*-SNE plots highlighted by each molecule of the CD4^{+} T cells. Circles indicate FoxP3^{+}CD4^{+} Treg cells. n.s., non-significant.

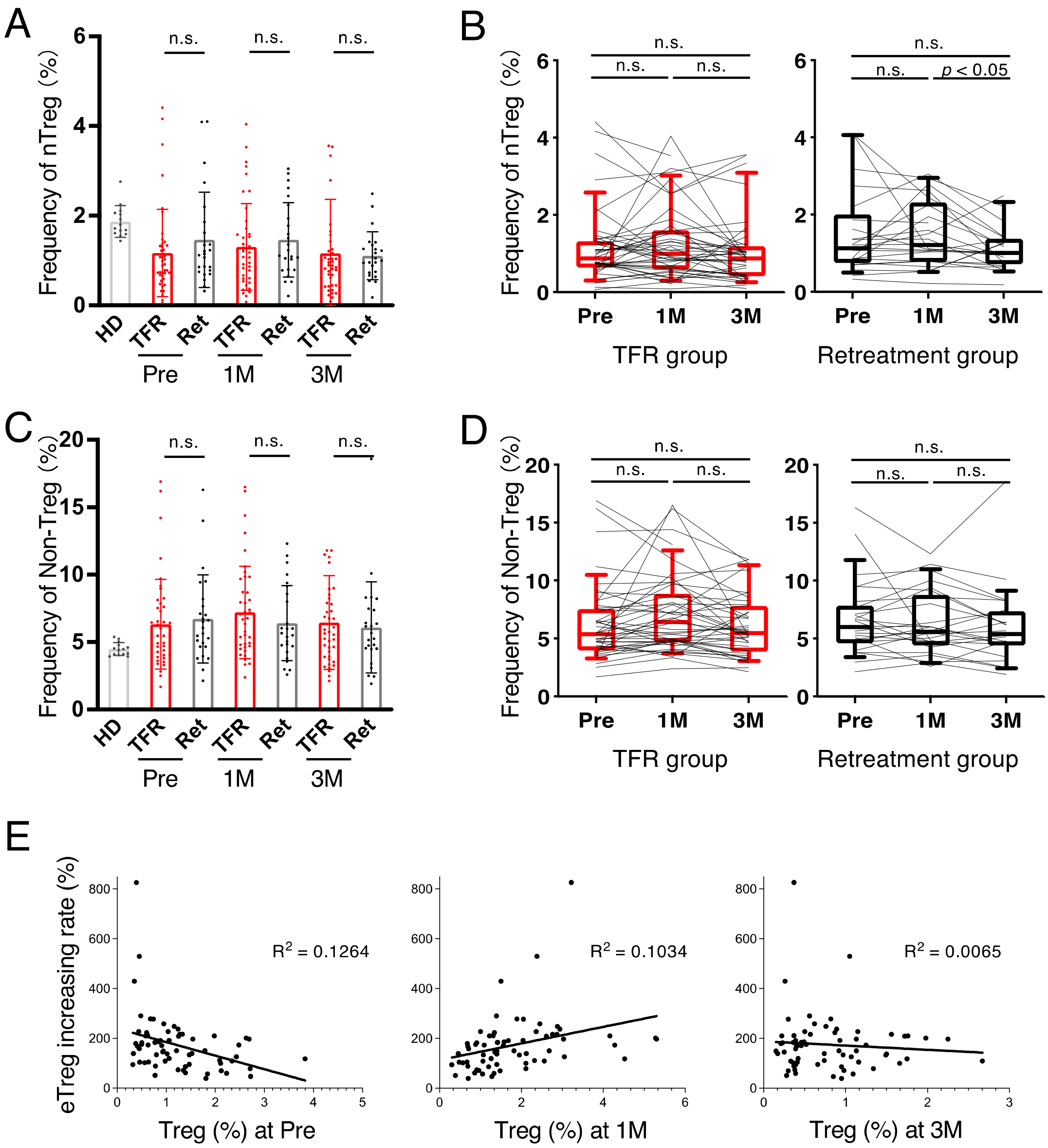

Figure S3. The frequency of the naïve Treg (nTreg) and non-Treg cells showed no difference between the TFR and retreatment (Ret) groups at Pre, 1M, and 3M. (**A**) Frequencies of CD45RA$^+$FoxP3$^+$CD4$^+$ nTreg cells among CD4$^+$ T cells in a HD and in patients with CML from the TFR (red) and Ret (black) groups. (**B**) Kinetics of nTreg cells in patients with CML from TFR and retreatment groups. (**C**) Frequencies of CD45RA$^-$FoxP3^{low}CD4$^+$ non-Treg cells among CD4$^+$ T cells in HD and CML patients from TFR (red) and Ret (black) groups. (**D**) Kinetics of non-Treg cells in CML patients from TFR and retreatment groups. (**E**) Correlation between eTreg increasing ratio and the frequency of eTreg at Pre, 1M, and 3M. n.s., non-significant. R, correlation coefficients.

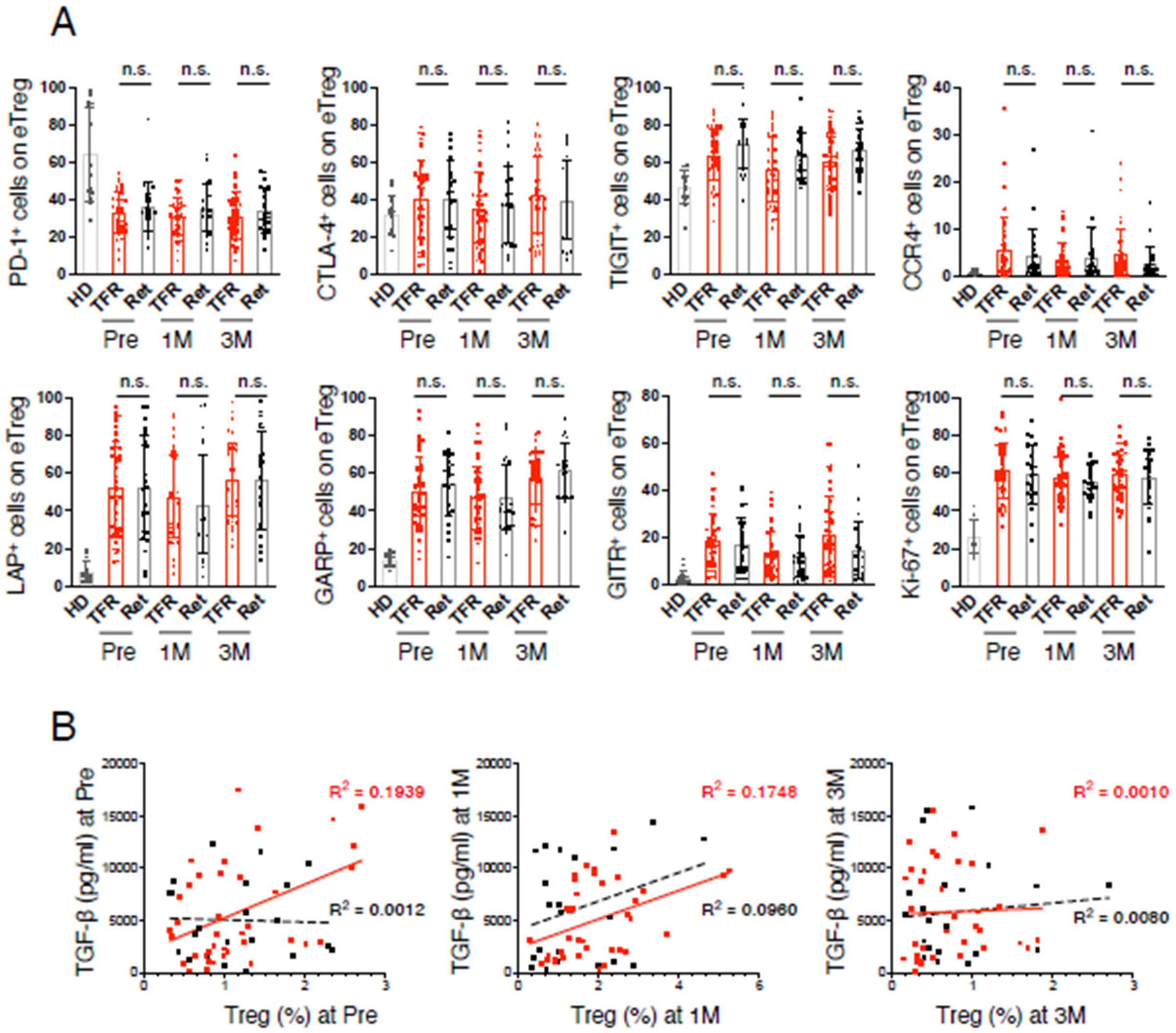

Figure S4. Treg cell phenotypes did not differ between the TFR and retreatment (Ret) groups at Pre, 1M, and 3M. (**A**) Frequencies of several co-stimulatory molecules and Ki-67+ eTreg cells in a HD and in patients with CML from the TFR (red) and Ret (black) groups. (**B**) Correlation between the serum levels of TGF-beta and the frequency of eTreg at Pre, 1M, and 3M. Red and black dotted lines indicate the regression lines for TFR and retreatment groups, respectively. n.s., non-significant. R, correlation coefficient.

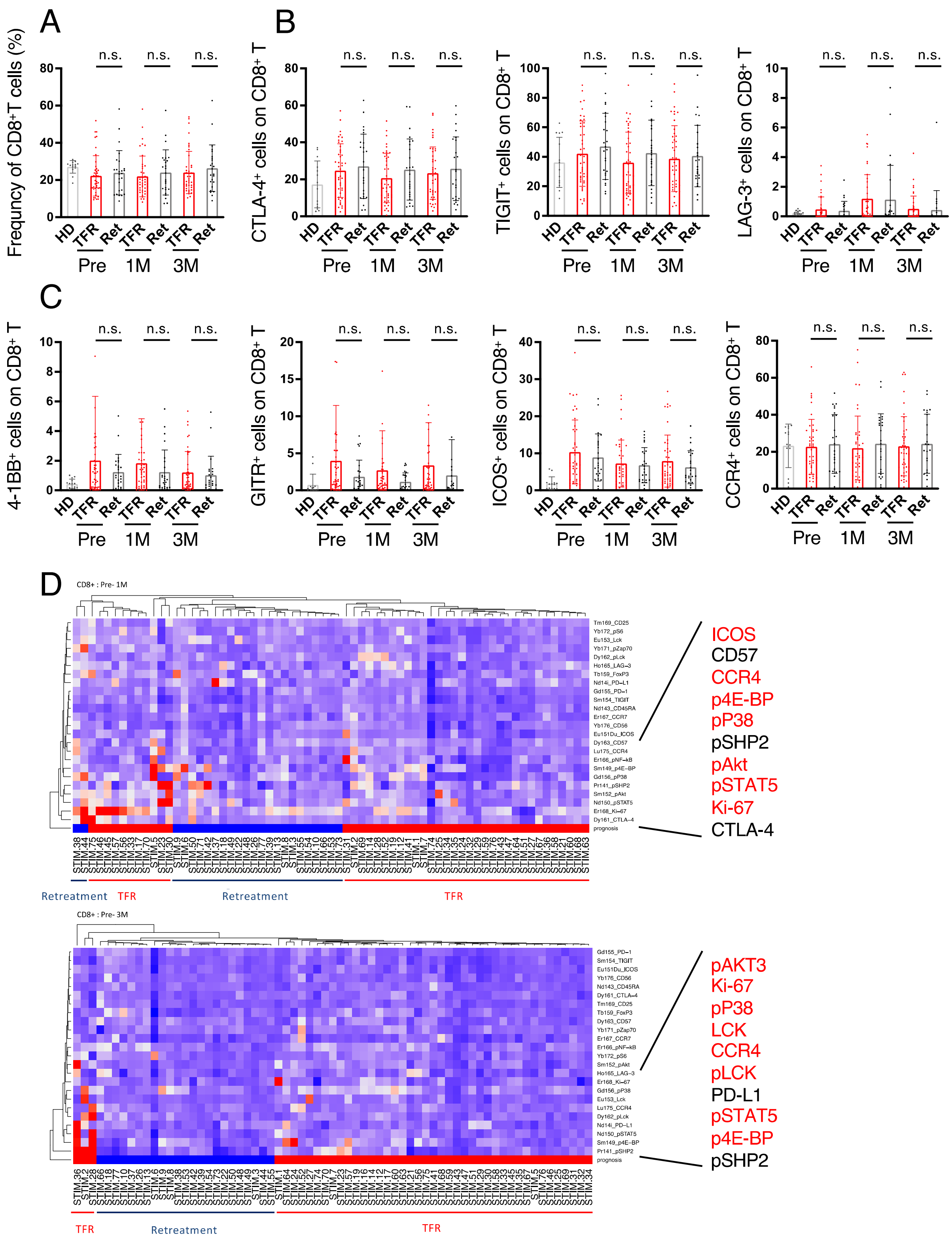

Figure S5. Phenotype of CD8+ T cells in an HD and in patients with CML at Pre, 1M, and 3M. (**A**) Frequencies of CD8+ T cells among CD3+ T cells in an HD and in CML patients from TFR (red) and retreatment (Ret) (black) groups. (**B**) Frequencies of several inhibitory co-stimulatory molecules positive CD8+ T cells in an HD and in CML patients from TFR (red) and Ret (black) groups. (**C**) Frequencies of several promotive co-stimulatory molecules positive CD8+ T cells in an HD and in CML patients from TFR (red) and Ret (black) groups. (**D**) Heat map analyzed by hierarchical cluster indicated the correlation between the prognosis after stopping imatinib and intracellular signaling molecules measured using mass cytometry. Color tone shows the expression change of each molecule in CD8+ T cells from pre-stopping to 1 month (upper) or 3 months (lower) after stopping imatinib. Listed molecules in X-axis indicate that the lower the position the description, the more corelates with prognosis, because lowermost red or blue indicate the patients with TFR or retreatment, respectively. Red molecules generally function to activate cells. n.s., non-significant.

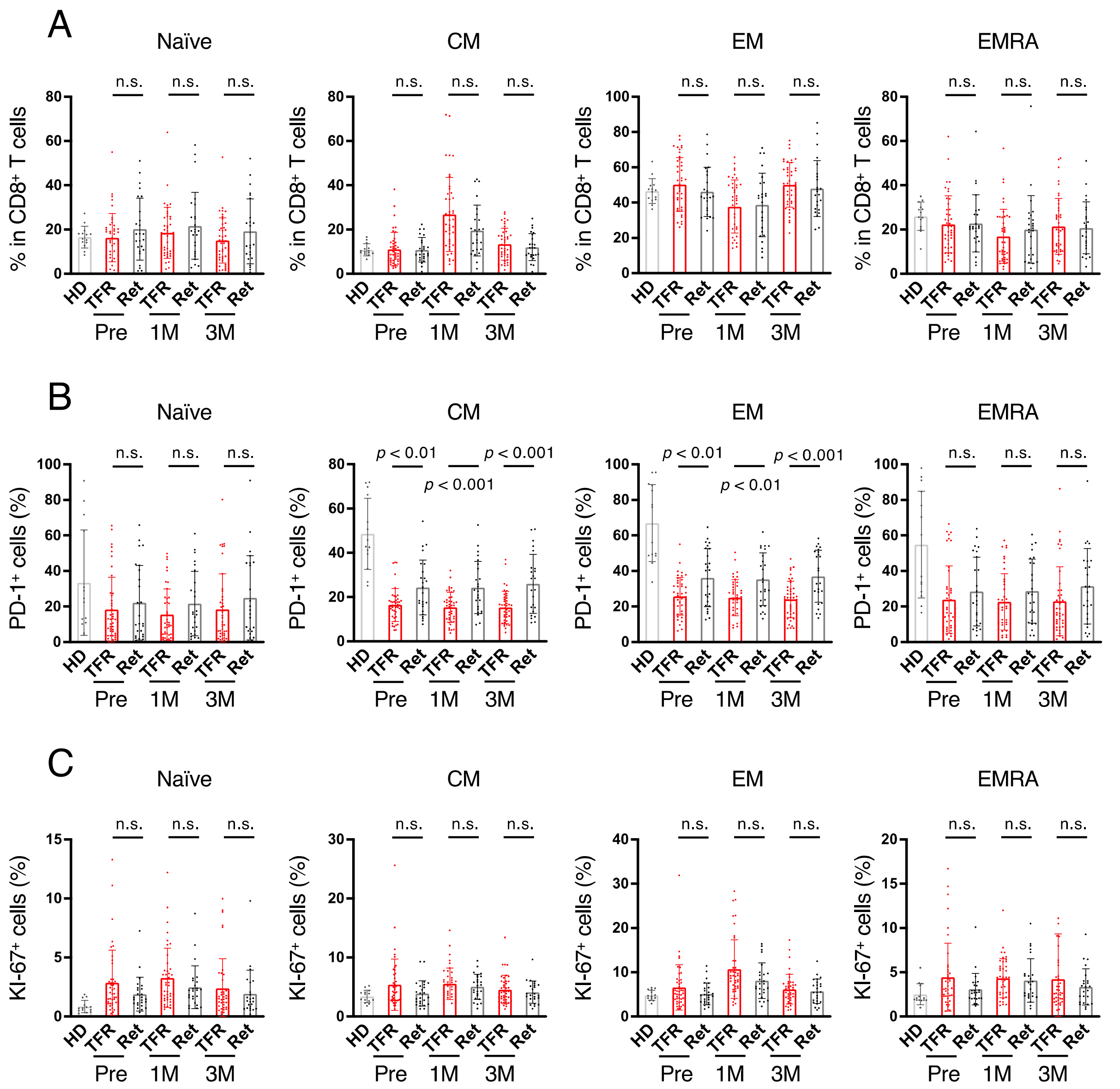

Figure S6. PD-1 expression in the central memory (CM) or effector memory (EM) $CD8^+$ T cells was significantly decreased in the TFR group. (**A**) Percentage of naïve, CM, EM, and $CD45RA^+$ EM (EMRA) $CD8^+$ T cells in an HD and in patients with CML from TFR (red) and retreatment (Ret) (black) groups at Pre, 1M, and 3M. (**B**) PD-1 positivity in naïve, CM, EM and EMRA $CD8^+$ T cells from HD and CML patients of TFR (red) and Ret (black) groups. (**C**) Ki-67 positivity in naïve, CM, EM and EMRA $CD8^+$ T cells in an HD and in CML patients from TFR (red) and Ret (black) groups. n.s., non-significant.

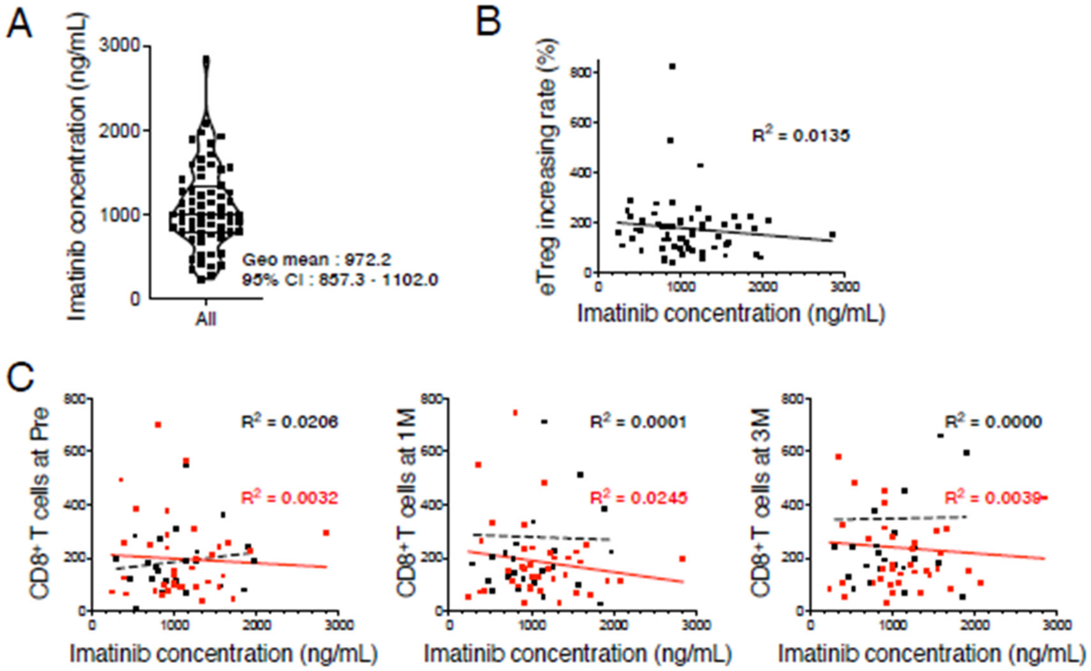

Figure S7. High imatinib concentration was inversely correlated to the frequency of CD8+ T cells of the TFR group after 1 month of stopping imatinib. (**A**) Plasma concentrations of imatinib in all patients measured using liquid chromatography before stopping imatinib administration. (**B**) Correlation between eTreg increasing ratio and imatinib trough concentration. R, correlation coefficient. (**C**) Correlation between the frequency of CD8+ T cells and imatinib trough concentration at Pre, 1M, and 3M. Red and black dotted lines indicate the regression lines of TFR and retreatment groups, respectively. R, correlation coefficients.

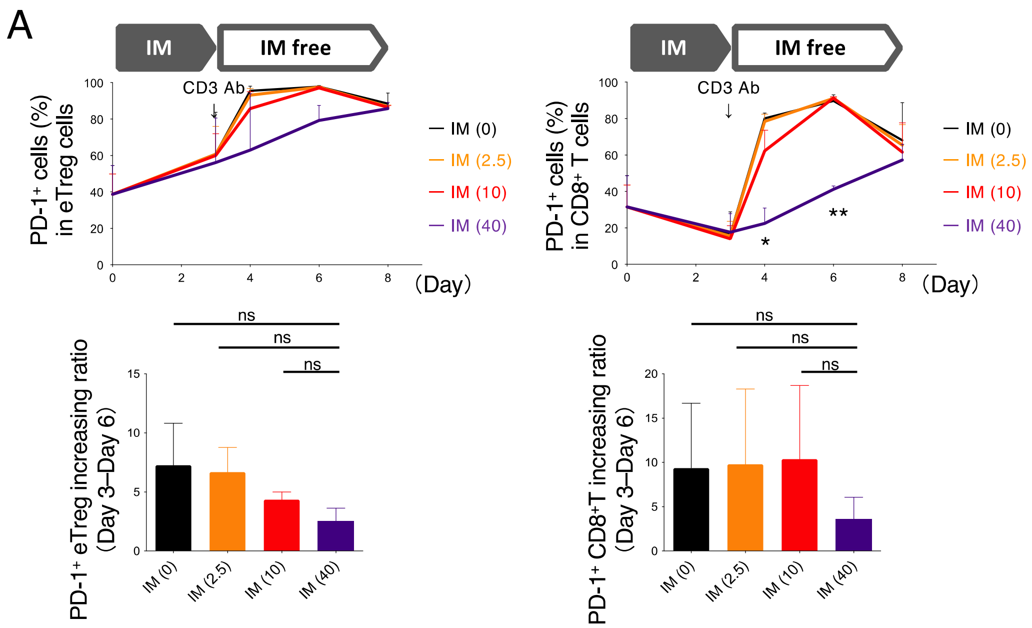

Figure S8. Peripheral blood mononuclear cells (PBMCs) from healthy donors were cultured with/without imatinib. The media containing imatinib were washed out and stimulated with anti-CD3 antibody on day 3. Cultured cells were sampled on day 0, 3, 4, 6, and 8 and subjected to flow cytometry. The course of PD-1 positivity in eTreg cells (left) and CD8+ T cells (right) (upper). Bar-graph shows the increase of PD-1+ eTreg cells (left) and PD-1+ CD8+ T cells (right) from day 3 to day 6 (lower). ns, not significant.

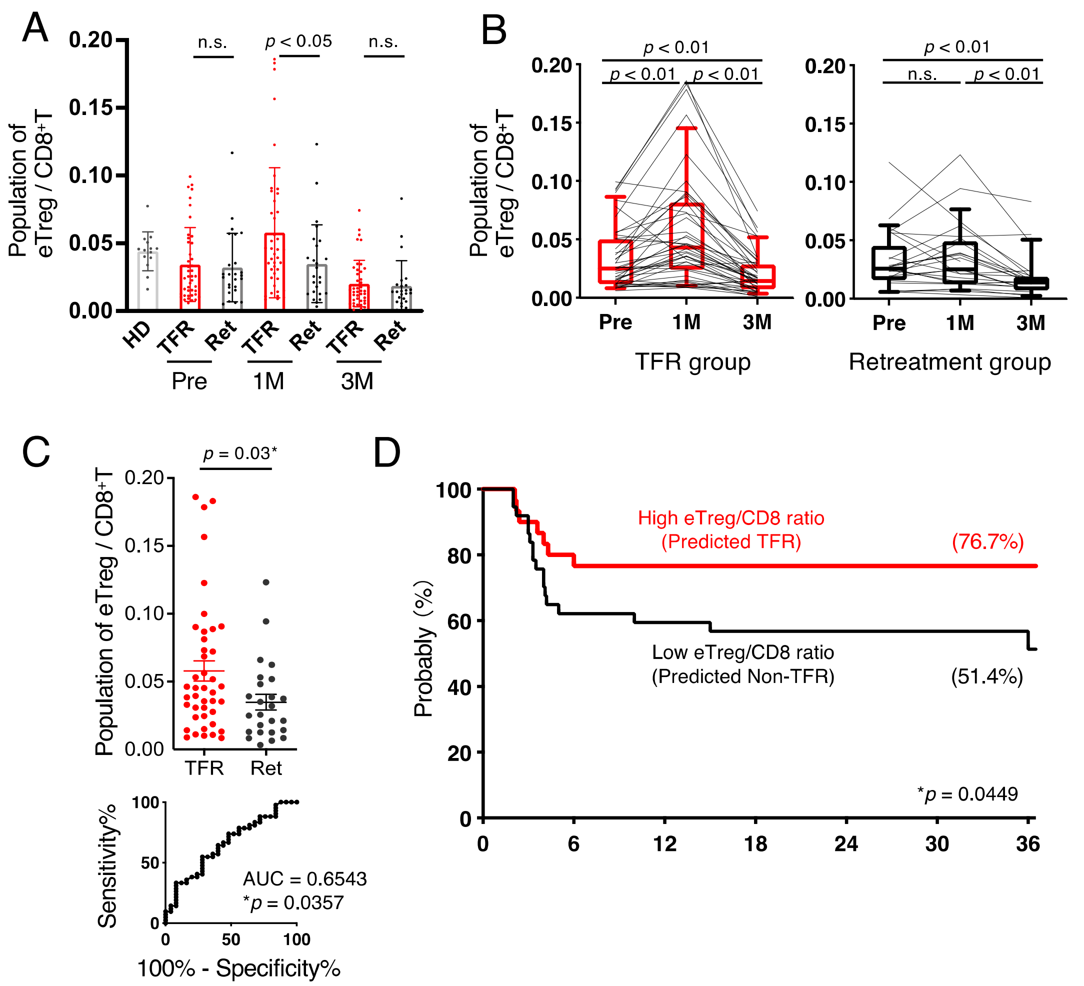

Figure S9. The combination of eTreg cells and CD8^{+} T cells could predict the maintenance of TFR. (**A**) Population of eTreg/CD8^{+}T in a HD and in patients with CML from the TFR (red) and retreatment (Ret) (black) groups at Pre, 1M, and 3M. (**B**) Kinetics of eTreg/CD8^{+}T ratio in CML patients from TFR and retreatment groups. (**C**) Scatter plot of eTreg/CD8^{+}T ratio at 1 month after stopping (top) and ROC curve for eTreg:CD8^{+}T ratio (bottom). (**D**) Kaplan–Meier curve for TFR rate validated by eTreg/CD8^{+}T ratio. Cut-off value of eTreg increasing ratio was calculated using the Youden index. n.s., non-significant. * $p < 0.05$.

Table S1. Antibodies used for mass cytometry.

Molecule	Clone	Company	Conjugation
pSHP2	D66F10	Fluidigm	141Pr
CD19	HIB19	Fluidigm	142Nd
CD45RA	HI100	Fluidigm	143Nd
CD11b	ICRF44	Fluidigm	144Nd
CD8a	RPA-T8	Fluidigm	146Nd
CD11c	Bu15	Fluidigm	147Sm
PD-L1	29E.2A3	Fluidigm	148Nd
p4E-PB1	236B4	Fluidigm	149Sm
pSTAT5	47	Fluidigm	150Nd
ICOS	C398.4A	Fluidigm	151Eu
pAkt	D9E	Fluidigm	152Sm
Lck	LCK-01	Fluidigm	153Eu
TIGIT	MBSA43	Fluidigm	154Sm
PD-1	EH12.2H7	Fluidigm	155Gd
pP38	D3F9	Fluidigm	156Gd

CD33	WM53	Fluidigm	158Gd
FoxP3	236A/E7	Fluidigm	159Tb
CD14	MSE2	Fluidigm	160Gd
CTLA-4	14D3	Fluidigm	161Dy
pLck	4/LCK-Y505	Fluidigm	162Dy
CD57	HCD57	Fluidigm	163Dy
LAG-3	17B4	Fluidigm	165Ho
pNFkBp65	K10x	Fluidigm	166Er
CCR7	G043H7	Fluidigm	167Er
Ki-67	Ki67	Fluidigm	168Er
CD25	2A3	Fluidigm	169Tm
CD3	UCHT1	Fluidigm	170Er
pS6	N7-548	Fluidigm	172Yb
HLA-DR	L243	Fluidigm	173Yb
CD4	SK3	Fluidigm	174Yb
CCR4	1G1	Fluidigm	175Lu
CD56	NCAM16.2	Fluidigm	176Yb
CD16	3G8	Fluidigm	209Bi

Table S2. Antibodies used for flow cytometry.

Molecule	Clone	Company	Conjugation
CD3	UCHT1	BD Biosciences	AF-700
CD4	RPA-T4	BioLegend	BV510
CD8a	RPA-T8	BioLegend	BV785
CD45RA	HI100	BioLegend	BV711
FoxP3	236A/E7	BD Biosciences	PE
CCR7	G043H7	BioLegend	BV605
CCR4	1G1	BD Biosciences	PerCP-Cy5.5
PD-1	EH12.2H7	BioLegend	BV421
CTLA-4	L3D10	BioLegend	PE-Cy7
LAG-3	17B4	ENZO	FITC
ICOS	C398.4A	BD Biosciences	PE/Dazzle 594
GITR	eBioAITR	Thermo Fisher Scientific	PE-eFluor610
TIGIT	BMSA43	Thermo Fisher Scientific	APC
4-1BB	4B4-1	BioLegend	PE-Cy7
CD27	O323	BioLegend	FITC
Tim-3	F38-2E2	BioLegend	PE-Dazzle
Ki-67	CD28.2	Thermo Fisher Scientific	PerCP-eFluor710
GARP	G14D9	Thermo Fisher Scientific	eFluor660
LAP	FNLAP	Thermo Fisher Scientific	PE-Cy7
OX40	ACT35	BioLegend	FITC
CD16	B73.1	BioLegend	PE-Cy7
CD56	HCD56	BioLegend	PerCP-Cy5.5
CD57	NK-1	BD Biosciences	PE-CF594
